# Genistein interferes with SDF-1- and HIV-mediated actin dynamics and inhibits HIV infection of resting CD4 T cells

**DOI:** 10.1186/1742-4690-10-62

**Published:** 2013-06-19

**Authors:** Jia Guo, Xuehua Xu, Taban K Rasheed, Alyson Yoder, Dongyang Yu, Huizhi Liang, Fei Yi, Todd Hawley, Tian Jin, Binhua Ling, Yuntao Wu

**Affiliations:** 1 National Center for Biodefense and Infectious Diseases, Department of Molecular and Microbiology, George Mason University, Manassas VA 20110, USA; 2 Chemotaxis Signal Section, Laboratory of Immunogenetics, National Institute of Allergy and Infectious Disease, NIH, Twinbrook Facility, Rockville, MD 20852, USA; 3 Division of Comparative Pathology, Tulane National Primate Research Center, Tulane University Health Sciences Center, Covington, LA 70433, USA

**Keywords:** HIV-1, Actin, SDF-1, Genistein, Sunitinib, Cofilin, LIMK1

## Abstract

**Background:**

Binding of HIV to the chemokine coreceptor CXCR4 mediates viral fusion and signal transduction that promotes actin dynamics critical for HIV infection of blood resting CD4 T cells. It has been suggested that this gp120-mediated actin activity resembles the chemotactic actin dynamics mediated by chemokines such as SDF-1. To determine whether inhibiting SDF-1-mediated chemotactic activity can also inhibit HIV infection, we screened several inhibitors known to reduce SDF-1-mediated chemotaxis of T cells.

**Results:**

We found that a tyrosine kinase inhibitor, genistein, inhibited both SDF-1-mediated chemotaxis and HIV infection of resting CD4 T cells. Genistein was also found to interfere with SDF-1- and HIV-mediated actin dynamics in CD4 T cells. This reduction in actin activity correlates with genistein-mediated inhibition of viral DNA accumulation in resting CD4 T cells. In addition, we also tested two other tyrosine kinase inhibitors, sunitinib and AG1478. Sunitinib, but not AG1478, inhibited HIV infection of resting CD4 T cells. We further tested the safety of genistein in 3 Chinese rhesus macaques (*Macaca mulatta*), and each animal was given a monotherapy of genistein at 10 mg/kg orally for 12 weeks. No adverse drug effects were observed in these animals.

**Conclusions:**

Our results suggest that novel therapeutic strategies can be developed based on targeting cellular proteins involved in HIV-dependent signaling. This approach can interfere with HIV-mediated actin dynamics and inhibit HIV infection.

## Background

Binding of HIV to CD4 and the chemokine coreceptor CXCR4 or CCR5 mediates viral fusion and entry [1-8]. This interaction also triggers the activation of signaling molecules [9-11]. In particular, HIV binding to CXCR4 activates actin regulators such as LIMK1 and cofilin, promoting actin dynamics necessary for viral infection of resting T cells [12-16]. *In vivo*, chemokine receptor signaling promotes actin dynamics for chemotactic cell migration. Consistently, induction of actin activity by treatment of resting CD4 T cells with chemokines such as CCL2 augments gp120-induced F-actin polymerization and enhances viral DNA synthesis [17]. Similar treatment of memory CD4 T cells with CCL19 triggers cofilin activation and changes in actin filaments, which greatly promote viral nuclear localization and DNA integration [18-20]. In addition, spinoculation of CD4 T cells triggers both cofilin activation and actin dynamics, leading to a great enhancement of HIV DNA synthesis and nuclear migration [21].

Mechanistically, HIV-mediated actin dynamics are involved in viral early steps, such as entry, post-entry DNA synthesis, and nuclear migration. During viral entry, HIV-1 binding to resting CD4 T cells triggers a rapid and transient actin polymerization through Rac1-PAK1/2-LIMK1-cofilin activation [14]. This rapid actin polymerization transiently blocks CXCR4 internalization to prolong gp120-CXCR4 interaction for membrane fusion to occur [14]. Other actin binding proteins such as Arp2/3, filamin-A, and moesin may also promote actin polymerization and anchor F-actin to membrane proteins to facilitate receptor clustering and viral fusion [22-26]. Following viral entry, the viral reverse transcription complex may be anchored onto F-actin [27-34] for optimal reverse transcription [12,14,35]. In addition, HIV-mediated actin treadmilling through CXCR4 signaling and cofilin activity promotes HIV intracellular migration and nuclear localization [12].

To some extent, HIV-mediated signal transduction through CXCR4 resembles the chemotactic response mediated by chemokines such as SDF-1, the natural ligand for CXCR4 [36,37]. The major biological response of SDF-1/CXCR4 interaction is to trigger rapid actin activity, which drives directional cell migration. Given that both SDF-1 and HIV require actin dynamics for cell migration and infection, respectively, we speculated that inhibition of certain shared chemotactic pathways between SDF-1 and gp120 may also inhibit HIV infection of resting T cells. Thus, we tested multiple known chemotactic inhibitors of SDF-1 to determine whether these inhibitors can also inhibit HIV. We demonstrate that a tyrosine kinase inhibitor, genistein, known to inhibit SDF-1-mediated chemotaxis, inhibited HIV infection of resting T cells.

Genistein is a tyrosine kinase inhibitor found in a number of plants such as soybeans and flemingia vestita [38], and is being tested for treatment of cancers such as leukemia [39,40] and prostate cancer [41,42]. Genistein has been shown to inhibit human prostate cancer cell migration through inhibiting pro-motility signaling [43,44]. Genistein similarly inhibits SDF-1-mediated chemotaxis of CD4 T cells [45], and is suggested to modulate the cellular distribution of actin-binding proteins in human stromal cells by inducing the peri-nuclear accumulation of the actin-binding proteins formin-2 and profilin [46]. In addition, genistein has been shown to inhibit HIV infection of resting CD4 T cells [47,48] and macrophages by affecting an unknown early step at entry and post-entry [49]. These previous findings have led us to speculate that genistein may inhibit HIV infection of resting CD4 T cells through interference with HIV-mediated actin dynamics. In this report we demonstrate that genistein interferes with HIV-mediated actin dynamics and inhibits viral post-entry DNA synthesis and, to a lesser extent, viral DNA nuclear migration in resting T cells. Our results highlight the possibility that novel therapeutic strategies can be developed to target the HIV-mediated cellular signal transduction to actin dynamics.

## Results

### Genistein inhibits SDF-1-mediated chemotaxis and HIV infection of resting CD4 T cells

Given that both SDF-1 and HIV trigger rapid actin rearrangement in resting CD4 T cells [12,50], we asked whether chemotaxis inhibitors can also inhibit gp120-mediated chemotactic signaling and HIV infection of resting T cells. Indeed, the Gαi inhibitor pertussis toxin (PTX) has been shown to inhibit both SDF-1 and gp120-mediated actin dynamics, and HIV-1 infection of resting T cells [12]. Thus, we tested several known SDF-1 inhibitors including the tyrosine kinase inhibitors herbimycin and genistein, and the cyclic nucleotides 8-Br-cAMP and 8-Br-cGMP. These inhibitors have been previously shown to affect SDF-1-mediated memory CD4 T cell movement towards or away from SDF-1 [45].

**Figure 1:**
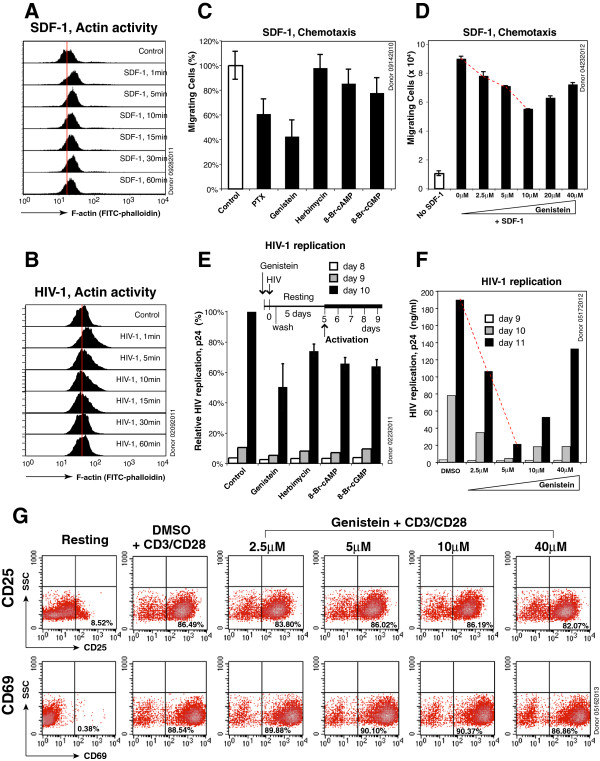
**Genistein inhibits SDF-1-mediated chemotaxis and HIV infection of resting CD4 T cells.** (**A**) Resting memory CD4 T cells were stimulated with SDF-1 (12.5 nM) for various times, stained with FITC-phalloidin, and then analyzed with flow cytometer. (**B**) Resting memory CD4 T cells were stimulated with HIV-1_NL4-3_ for various times and similarly stained with FITC-phalloidin and analyzed. (**C**) Resting memory CD4 T ells were pretreated with pertussis toxin (PTX, 100 ng/ml), genistein (3.7 μM), herbimycin (1 μM), 8-Br-cAMP (100 μM), 8-Br-cGMP (100 μM), or DMSO (1%, control) for 15–60 minutes, and then assayed for migration towards SDF-1 (12.5 nM). Results are expressed as the relative percentage of migrating cells. (**D**) Dosage-dependent inhibition of SDF-1-mediated chemotaxis by genistein. Experiments were carried out as described in (**C**) with memory T cells pretreated with different dosages of genistein. (**E**) Resting CD4 T cells were similarly pretreated with genistein, herbimycin, 8-Br-cAMP, 8-Br-cGMP, or DMSO, and infected with HIV-1_NL4-3_ for 2 hours in the continuous presence of these inhibitors. Following infection, cells were washed twice and then cultured in the absence of the inhibitors for 5 days. Cells were activated at day 5 with anti-CD3/CD28 magnetic beads (4 beads per cell), and viral replication was measured by p24 release. (**F**) Dosage-dependent inhibition of HIV-1 infection by genistein. Experiments were carried out as described in (**E**) with resting T cells pretreated with different dosages of genistein. (**G**) Effects of genistein on T cell activation. Resting CD4 T cells were treated with genistein at different dosages for 3 hours, washed twice, and then cultured in the absence of genistein as described in (**E**). Cells were similarly activated with anti-CD3/CD28 beads. At 24 hours after stimulation, cells were stained with PE-labeled monoclonal antibody against human CD25 or CD69, and then analyzed with flow cytometry.

We purified human resting CD45RO^+^/CD45RA^-^ memory CD4 T cells by negative selection (98% pure, measured by flow cytometry, data not shown) [51], and then similarly stimulated these cells with either SDF-1 or HIV-1_NL4-3_ for a time course. We measured SDF-1- and HIV-mediated actin dynamics, and observed quick actin polymerization both in SDF-1- and HIV-stimulated memory CD4 T cells, starting at 1 minute post treatment (Figure 1A and B). Next, we treated resting memory CD4 T cells with chemotactic inhibitors, including pertussis toxin (PTX), genistein, herbimycin, 8-Br-cAMP, or 8-Br-cGMP, and tested the inhibition of SDF-1-mediated chemotaxis in a chemotactic trans-well assay (Figure 1C). We observed reduction of memory T cell migration with PTX and genistein, consistent with previous results [45]. However, we did not observe significant inhibition of T cell chemotaxis in the trans-well assay by herbimycin, 8-Br-cAMP, or 8-Br-cGMP in this particular donor.

We also performed a genistein dosage-dependent assay of SDF-1-mediated chemotaxis, and observed dosage-dependent inhibition in concentrations from 2.5 to 10 μM (Figure 1D). However, at higher concentrations (20 and 40 μM), less inhibition was observed in this donor. Although genistein inhibited chemotaxis at all dosages, the overall relationship between inhibition and drug concentration was not linear. Given that genistein likely targets multiple tyrosine kinases which may antagonize each other, the result was not totally surprising, and may result from differing sensitivities of tyrosine kinases to genistein inhibition.

To determine whether these inhibitors can also inhibit HIV infection of resting CD4 T cells, we pretreated resting CD4 T cells with genistein, herbimycin, 8-Br-cAMP or 8-Br-cGMP, and then infected cells with HIV-1. Following infection for 2 hours, cell-free virus and the inhibitors were washed away, and cells were incubated in the absence of the inhibitors for 5 days, during which productive viral replication does not occur. However, viral replication is inducible upon CD3/CD28 stimulation [52]. As shown in Figure 1E, we activated infected cells with anti-CD3/CD28 beads and observed minimal inhibition of HIV replication by herbimycin, 8-Br-cAMP and 8-Br-cGMP. Nevertheless, we observed a 50% reduction of HIV replication by 3.7 μM genistein (at day 10) in this particular donor. We also performed an experiment on HIV-1 infection at different genistein dosages, and observed dosage-dependent inhibition in concentrations below 5 μM (Figure 1F). However, at higher dosages (10 and 40 μM), the inhibition were less in this donor, similar to the chemotaxis inhibition results in Figure 1D; although genistein inhibited HIV-1 replication at all dosages tested, the overall extent of inhibition was not strictly dosage-dependent. The inhibition of HIV infection did not result from cytotoxicity or inhibition of T cell activation by genistein; when resting CD4 T cells were similarly treated with genistein and activated with CD3/CD28 beads, we did not observe inhibition of T cells activation at all the dosages tested, as judged by the upregulation of the CD25 and CD69 surface receptors (Figure 1G).

### Genistein inhibits HIV infection of resting CD4 T cells, viral DNA synthesis, and viral nuclear migration

**Figure 2:**
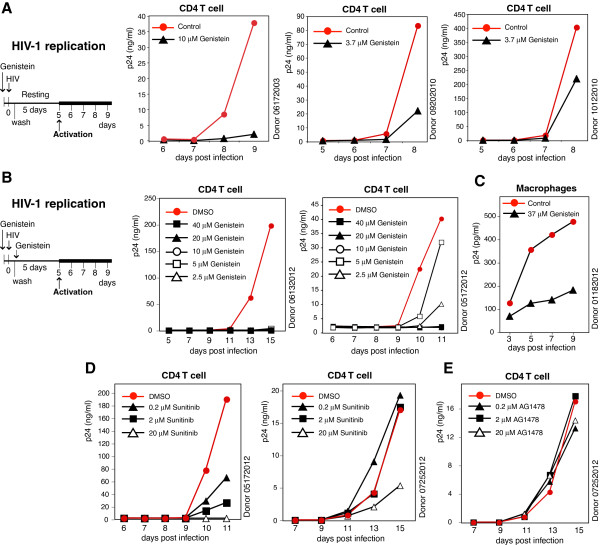
**Genistein inhibits HIV infection of resting CD4 T cells and macrophages.** (**A**) Genistein inhibits HIV infection of resting CD4 T cells in additional three donors. Experimental procedures are described in Figure 1E. (**B**) Dosage-dependent inhibition of HIV infection of resting CD4 T cells. Resting CD4 T cells from two additional donors were pretreated with genistein at different dosages for 1 hour, and then infected with HIV-1_NL4-3_ for 2 hours at 37°C in the continuous presence of these inhibitors. Following infection, cells were washed twice and a single dose of genistein was added to the culture. Cells were culture for 5 days and activated at day 5 with anti-CD3/CD28 magnetic beads (4 beads per cell). Viral replication was measured by p24 release. (**C**) Genistein inhibits HIV infection of primary macrophages. Human peripheral blood monocyte-derived macrophages were pretreated with genistein or DMSO (1%, control) for 1 hour, and then infected with HIV (THRO.c/2626) for 2 hours. Following infection, cells were washed to remove HIV and the inhibitor, and then continuously cultured in the absence of genistein to monitor viral replication. (**D**) Sunitinib inhibits HIV infection of resting CD4 T cells. Cells from two donors were pretreated with different dosages of sunitinib for 1 hour, and then infected with HIV-1_NL4-3_ for 2 hours in the continuous presence of the inhibitor. Following infection, cells were washed twice and then cultured in the absence of the inhibitor for 5 days. Cells were activated at day 5 with anti-CD3/CD28 magnetic beads, and viral replication was measured by p24 release. (**E**) AG1478 does not inhibit HIV infection of resting CD4 T cells. Resting CD4 T cells were similarly pretreated with AG1478, and infected with HIV-1, as described in (**D**).

To further confirm that genistein inhibits HIV infection of resting CD4 T cells, we repeated the above experiment (Figure 1E) in another 4 donors and observed inhibition of HIV infection by transient treatment of resting CD4 T cells with genistein during infection (Figure 2A and Additional file 1: Figure S1). Nevertheless, there were clear donor-dependent variations in the degree of inhibition (Figure 2A). We also performed genistein pre-treatment plus one-dose post-infection treatment of resting CD4 T cells, and observed complete inhibition of HIV at all concentrations tested (2.5 to 40 μM) in one donor. In a second donor, we also observed complete inhibition of HIV-1 at concentrations from 10 to 40 μM, and partial inhibition at 2.5 and 5 μM (Figure 2B and Additional file 1: Figure S2).

We further tested the effect of genistein on HIV infection of peripheral blood monocyte-derived macrophages. Cells were pretreated with 37 μM (or 10 μg/ml [49]) genistein for 1 hour and infected with a primary M-tropic HIV stain, THRO.c/2626 [53], for 2 hours. Following infection, both genistein and HIV were washed away, and viral replication was monitored. We observed inhibition of HIV by genistein (Figure 2C), similar to a previous report [49].

We also asked whether other clinical tyrosine kinase inhibitors would be able to inhibit HIV infection of resting T cells, and tested two anti-tumor drugs, sunitinib and AG1478 (tyrphostin). Sunitinib inhibits cellular signaling by targeting multiple receptor tyrosine kinases [54], whereas AG1478 selectively inhibits epidermal growth factor receptor (EGFR) activation by inhibiting EGFR tyrosine kinase [55]. As shown in Figure 2D, we observed inhibition of HIV-1 infection by sunitinib at 0.2-20 μM in one donor and at 20 μM in a second donor. We observed no inhibition of HIV-1 by AG1478 at all dosages tested (0.2-20 μM) in one donor.

**Figure 3:**
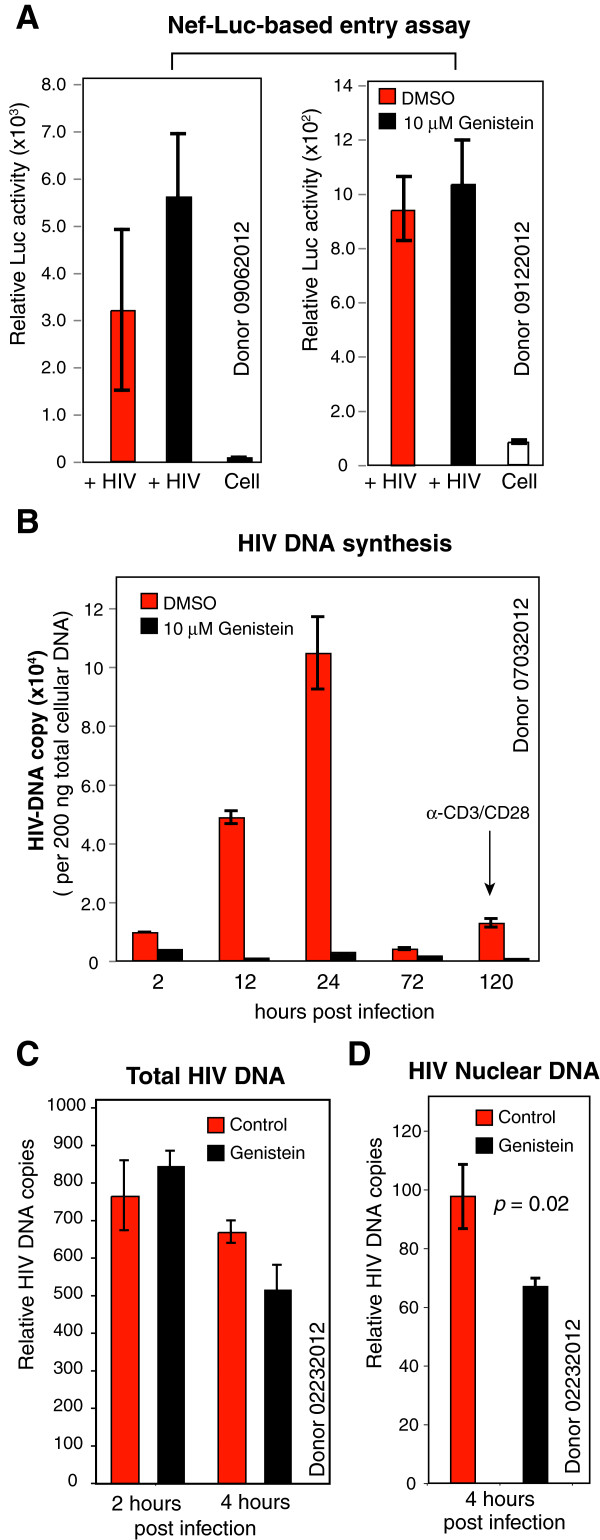
**Genistein inhibits HIV DNA synthesis and viral DNA nuclear localization.** (**A**) Genistein does not inhibit viral entry into resting CD4 T cells. Resting CD4 T cells from two donors were pretreated with genistein for 1 hour, and then infected with Nef-luciferase tagged HIV-1 for 2 hours. Cells were washed and luciferase activity was measured in live cells. (**B**) Genistein inhibits viral DNA synthesis in CD4 T cells. Resting memory CD4 T cells were pretreated with genistein (10 μM) or DMSO (1%, control) for 1 hour, and then infected with HIV-1_NL4-3_ for 2 hour in the presence of genistein. Cells were washed twice to remove HIV and genistein, cultured for 5 days, and then activated at day 5 with anti-CD3/CD28 magnetic beads. Infected cells were harvested and lysed at different time points following infection to extract total cellular DNA. HIV DNA was measured by using real time PCR using equal amount of total DNA. (**C** and **D**) Resting memory CD4 T cells were similarly pretreated with genistein (10 μM) and infected with HIV. Viral DNA was measured at 2 and 4 hours post infection. Infected cells were also used for fractionation and purification of nuclear DNA. HIV nuclear DNA was measured using real time PCR using equal amount of total DNA (**D**).

Previously, Stantchev *et al.* reported that 5–10 μg/ml (or 18.5-37 μM) genistein inhibited HIV infection of primary human macrophages [49]; genistein was also found to be non-toxic to cells for these several hours of short treatment at these dosages, and genistein also did not affect the surface expression of CD4 and CCR5 [49]. Interestingly, genistein blocked viral infection of macrophages if added to cells either before, at the time of infection, or immediately after infection, but not 24 hours later, suggesting that genistein-mediated inhibition is at the step of entry and early post-entry [49]. Thus, we also examined the early steps of HIV infection of resting memory CD4 T cells in the presence or absence of genistein. As shown in Figure 3A, we did not observe inhibition of viral entry using a Nef-luciferase based entry assay [56]. We then followed a time course of viral DNA synthesis. HIV reverse transcription in resting CD4 T cells is a biphasic slow process, with an early and a late DNA synthesis phase that peaks at 2–4 hours and 1–2 days respectively [12,57]. The process of viral DNA synthesis is also accompanied by viral DNA decay in the absence of chemotactic signaling to promote the nuclear entry of newly synthesized viral DNA [12,19,58]. As shown in Figure 3B, we observed that viral DNA synthesis peaked at day 1, and then decreased by day 3; in genistein-treated cells, viral DNA synthesis at day 1 was greatly inhibited. We also examined early viral nuclear entry (4 hours) which is promoted by HIV-1 gp120-CXCR4 signaling [12]. We observed a slight early decrease of viral nuclear DNA in genistein-treated cells (Figure 3D). In conclusion, our results suggest that genistein mainly inhibits the slow accumulation of viral DNA in resting CD4 T cells, and, to a lesser extent, viral nuclear migration. Our results are consistent with previous results on HIV infection of macrophages, suggesting that genistein affects early post-entry steps [49]. Although this previous study suggested that genistein may also inhibit viral entry in macrophages [49], we did not observe inhibition of viral entry in resting memory CD4 T cells using the Nef-luciferease entry assay (Figure 3A). The difference likely resulted from possible different modes of viral entry in these two different primary cell types. It has been shown that HIV can enter macrophages through membrane fusion and a macropinocytosis-like pathway [59], whereas in blood resting CD4 T cells, the endocytic entry pathway appears to be defective [13,60]. Genistein may have a different impact on viral entry into these two different cell types.

### Genistein interferes with SDF-1- and HIV-mediated actin dynamics in resting CD4 T cells

**Figure 4:**
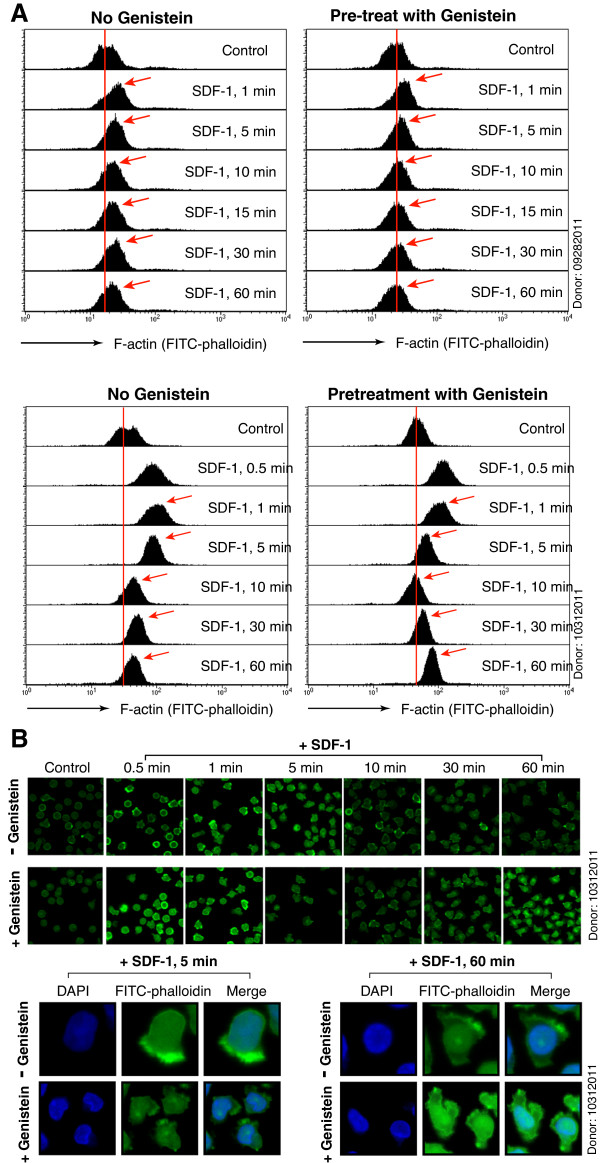
**Genistein interferes with SDF-1-mediated actin dynamics in resting memory CD4 T cells.** (**A**) Resting memory CD4 T cells from two donors were pretreated with genistein (3.7 μM) or mock-treated for 1 hour at 37°C, and then stimulated with SDF-1 (12.5 nM) for various times, from 0.5 minute to 60 minutes. Cells were fixed, stained with FITC-phalloidin for F-actin, and then analyzed with flow cytometer. (**B**) Confocal images of cells stained in (**A**)*,* showing F-actin staining. Images were acquired and analyzed at identical conditions for all samples. Some cells were also stained with DAPI (4′, 6-diamidino-2-phenylindole) for nuclear DNA.

Given that HIV-mediated actin dynamics play an important role in HIV infection of resting CD4 T cells [12,14,19], we speculated that genistein-mediated inhibition of HIV infection may be related to its inhibition of actin activity. The direct effect of genistein on T cell actin dynamics has not been studied although genistein inhibits SDF-1-mediated chemotaxis of memory CD4 T cells (Figure 1A to D) [45]. Genistein has been suggested to inhibit metastasis of cancer cells by inhibiting cell signaling and the redistribution of actin-binding proteins such as formin-2 and profilin [46]. Thus, we measured effects of genistein on SDF-1-mediated actin dynamics in resting memory CD4 T cells which were pre-treated with 3.7 μM genistein for 1 hour. This dosage of genistein slightly increased basal levels of actin density in some donors but not the others (data not shown). Following genistein pre-treatment, cells were treated with SDF-1 for a time course and actin dynamics were measured. As shown in Figure 4A, genistein did not inhibit SDF-1-mediated early actin polymerization (1 min), but it triggered a faster actin depolymerization at later times (after 5 min), which reduced the sustainability of actin polymerization, decreasing the overall actin dynamics. Similar results were observed in another donor (Additional file 1: Figure S3). Confocal microscopy of genistein-treated cells revealed that there was no gross alteration of cell morphology by genistein, but at 60 minutes after SDF-1 treatment, genistein also appeared to increase nuclear actin accumulation in this particular donor (Figure 4B and Additional file 1: Figure S4).

**Figure 5:**
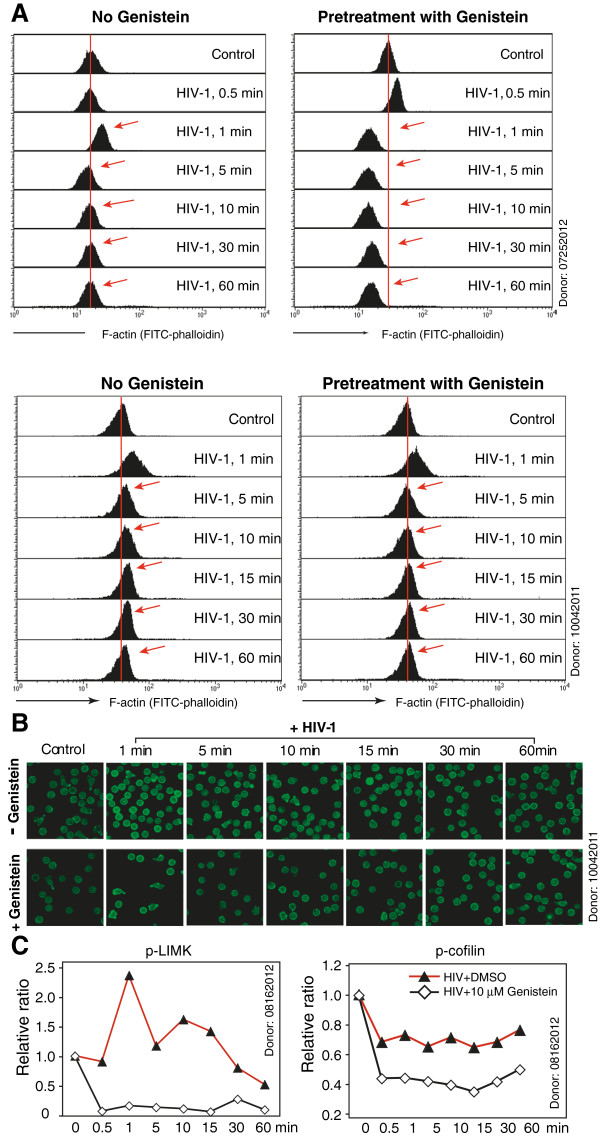
**Genistein interferes with HIV-mediated actin dynamics in resting memory CD4 T cells.** (**A**) Resting memory CD4 T cells from two donors were pretreated with genistein (3.7 μM) or mock-treated for 1 hour at 37°C, and then stimulated with HIV-1_NL4-3_ (100 ng) for various times. Cells were fixed, stained with FITC-phalloidin for F-actin, and then analyzed with a flow cytometer. (**B**) Confocal images of cells stained in (**A**)*,* showing F-actin staining. Images were acquired and analyzed at identical conditions for all samples. (**C**) Resting memory CD4 T cells were pretreated with 10 μM genistein for 1 hour, and then treated with HIV-1_NL4-3_ for a time course. Cells were then analyzed by Western blot for LIMK and cofilin activation, using an anti-phospho-LIMK antibody or an anti-cofilin antibody. The same blots were also probed with an anti-human GAPDH antibody for loading control and normalization.

To determine whether genistein similarly affects HIV-mediated actin dynamics, we pre-treated resting memory CD4 T cells with genistein, infected cells with HIV-1, and then measured virus-mediated actin dynamics. As shown in Figure 5A and B, we observed a similar pattern as seen in Figure 4A. Again, genistein did not inhibit HIV-mediated early actin polymerization (0.5-1 minute), but promoted faster actin depolymerization, reducing the overall actin activity (Additional file 1: Figure S5). We also performed Western blots to examine effects of genistein on actin regulators such as the LIM domain kinase (LIMK) and cofilin [12,14], which mediate actin depolymerization [61]. As shown in Figure 5C, we observed that genistein decreased HIV-1-mediated LIMK and cofilin phosphorylation and activation, disrupting the signaling pathway. Nevertheless, the effect is likely indirect, possibly resulting from inhibition of upstream tyrosine kinases, as LIMK1/2 and cofilin are phosphorylated on threonine 508/505 and serine 3, respectively [62,63].

### Pre-stimulation of CD4/CXCR4 receptors diminishes genistein-mediated inhibition of HIV infection of resting CD4 T cells

**Figure 6:**
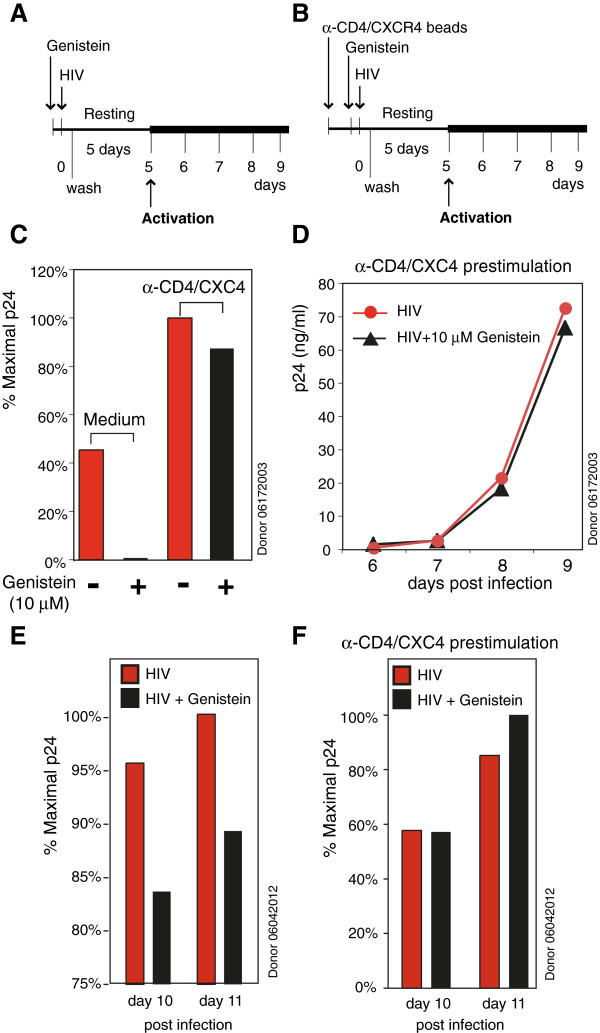
**Anti-CD4/CXCR4 bead pre-stimulation abolishes genistein inhibition of HIV infection of resting CD4 T cells.** (**A** and **B**) Purified resting CD4 T cells were cultured in medium overnight (**A**) or pre-stimulated with anti-CD4/CXCR4 magnetic beads (2 beads per cell) overnight (**B**), and then treated with genistein (10 μM) for 1 hour. Cells were infected with HIV-1_NL4-3_ for 2 hours, washed, incubated for 5 days in the absence of genistein, and then activated by anti-CD3/CD28 magnetic beads (2 beads per cell). Viral replication was measured by p24 release into the supernatant. (**C**) Viral replication measured at day 8 post infection. (**D**) The full viral replication course in cells pre-stimulated with anti-CD4/CXCR4 beads. (**E** and **F**) A repeat of the experiment in another donor. Viral replication at day 10 and 11 is shown.

Genistein is a general tyrosine kinase inhibitor, and it is likely that genistein indirectly affected SDF-1- and HIV-1-mediated actin dynamics through inhibition of tyrosine kinases that are involved in actin dynamics. Previously, we have demonstrated that pre-stimulation of resting CD4 T cells with anti-CD4/CXCR4 beads triggers cell signaling and actin reorganization that enhances HIV-1 infection of resting T cells [12]. This low-level stimulation (two beads per cell) does not block HIV entry or activate T cells, but greatly promotes actin dynamics that eliminate the inhibitory effect of the actin modulator jasplakinolide (at 120 and 600 nM) on HIV infection of resting T cells [12]. We speculated that similar stimulation of actin dynamics in resting CD4 T cells with anti-CD4/CXCR4 beads may also overcome genistein inhibition of HIV infection. As expected, we observed a complete loss of the inhibition by genistein when cells were pre-stimulated with anti-CD4/CXCR4 beads (Figure 6A to D), whereas in unstimulated cells, genistein inhibited over 99% of viral replication in CD4 T cells of the same donor (at day 8) (Figure 6C and D). Similar results were observed in another donor, although this donor demonstrated less inhibition of HIV-1 by 10 μM genistein (Figure 6E and F). These results are consistent with our hypothesis that genistein inhibits HIV infection through interference of the CD4/CXCR4-mediated receptor signaling that leads to actin dynamics.

### Genistein safety evaluation in rhesus macaques

**Figure 7:**
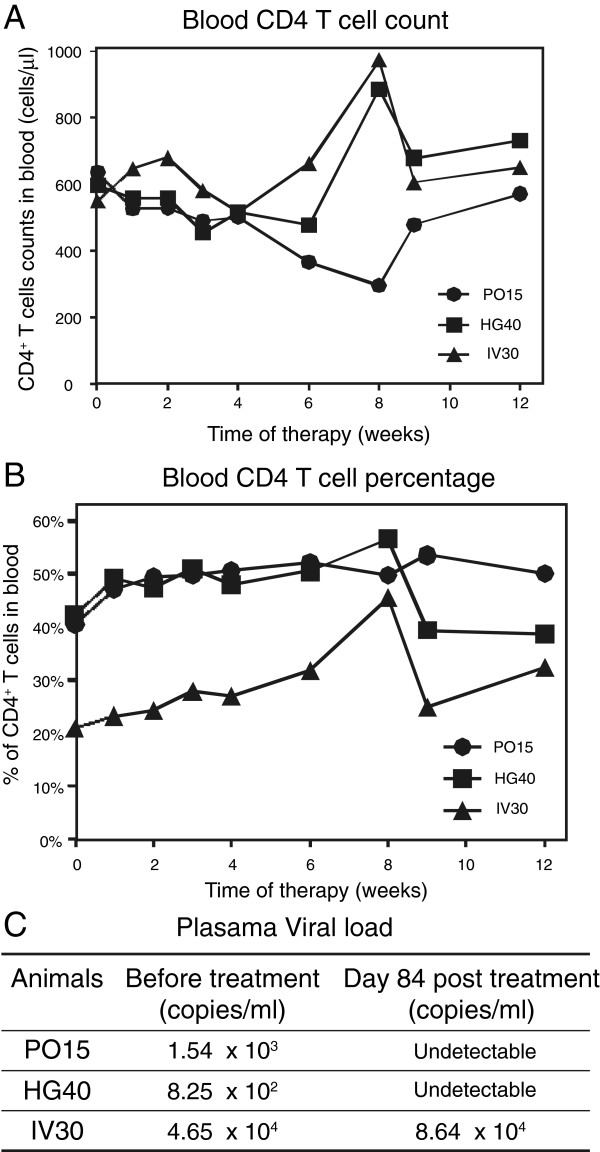
**Genistein monotherapy on Chinese rhesus macaques with chronic SIV infectionc.** (**A** and **B**) Three Chinese rhesus macaques were chronically infected with SIVmac251. Each animal received 10 mg/kg of genistein orally for 12 weeks. The dynamics of the absolute CD4+ T cell count (**A**) and the percentages of CD4+ T cells (**B**) in peripheral blood were monitored. The percentage of CD4+ T cells were calculated by gating on total CD3+ T lymphocytes analyzed by flow cytometry. The absolute CD4+ T cell counts were generated from the calculation of percentages of CD4+ T cells, CD3+ T cells and lymphocytes from the complete blood count (CBC). (**C**) Plasma viral load before and after genistein monotherapy was also measured.

Genistein is naturally made in a number of plants, such as soybeans, and its consumption is associated with a lower incidence of metastatic prostate cancer in southeast Asians who subsist on a soybean-based diet [41,64-67]. The average steady-state blood levels of genistein in Japanese men, who subsist on a soy-based diet, were 0.28 μM [66]. In a phase I human clinical trial, subjects were given genistein (43-90% pure) at 2–8 mg/kg orally, and no significant cytotoxicities were observed among these cancer patients [42]. The subjects sustained a maximal total plasma genistein concentration between 4.3 to 16.3 μM, with a drug half-life of 15 to 22 hours [42]. Based on this phase I study, we also initiated a preliminary animal trial to evaluate the safety of daily administration of genistein. Three rhesus macaques of Chinese-origin (*Macaca mulatta*) were chronically infected with SIVmac251 with plasma viral loads between 10^2^ to 10^4^ copies/ml. Each animal was given a monotherapy of genistein at 10 mg/kg orally for 12 weeks. We did not observe adverse effects on any of these animals, and their CD4 T cell counts and percentages remained stable in these 12 weeks (Figure 7A and B). We also performed a one-time measurement of plasma viral load at the end of the 12 weeks. Two of the animals (PO15 and HG40) had a reduction of viral load to undetectable level, whereas the third animal (IV30) had no reduction (Figure 7C). Given the donor variations and dosage-independent inhibition of HIV observed in our *in vitro* CD4 T cell cultures, IV30 may need longer treatment or a different genistein dosage. However, we do not excluded that drug resistance might also develop, although it is expected to be more difficult for cellular targets. Further studies are certainly needed to address the *in vivo* efficacy of genistein, and to define optimal dosages for maximal viral inhibition in individual animals.

## Discussion

In this report, we demonstrated that the T cell chemotaxis inhibitor, genistein, interfered with SDF-1-mediated actin dynamics. Similar treatment of resting T cells with genistein also interfered with HIV-1-mediated actin activity and inhibited HIV infection of resting T cells. Genistein is a tyrosine kinase inhibitor found in a number of plants such as soybeans and flemingia vestita [38], and is being tested for treatment of cancers such as leukemia [39,40] and prostate cancer [41,42]. Dietary genistein has also been shown to inhibit metastasis of human prostate cancer in mice [41,42]. Genistein inhibits human prostate cancer cell motility through inhibiting pro-motility signaling, specifically, by inhibiting the activation of FAK and the p38 MAPK-HSP27 pathway [43,44]. Genistein has also been suggested to modulate the cellular distribution of actin-binding proteins in human stromal cells by inducing the peri-nuclear accumulation of the actin-binding protein formin-2 and profilin [46]. Although the exact signaling molecules targeted by genistein in HIV infection were not systematically investigated in our study, genistein was found to inhibit the overall phosphorylation of LIMK and cofilin (Figure 5C), two of the main actin regulators involved in T cell mobility and HIV infection [12,14]. In cells, multiple actin regulatory proteins, such as gelsolin [68,69], villin [70], ezrin [71], cortactin [72-74], Rac1 [75], and WASP [76], require tyrosine phosphorylation for activity. Certainly, some of these molecules such as ezrin [23,24], Rac1 [14,77], and WASP [25,26] have also been implicated in HIV infection of CD4 T cells. It is possible that genistein may additionally affect these actin regulators leading to the inhibition of HIV-1 replication in resting CD4 T cells.

Our results on genistein-mediated reduction of both actin activity and HIV infection are consistent with a demonstrated role of early actin dynamics in HIV infection of resting T cells [12,14,17,19,51]. Our findings are also consistent with previous studies showing that chemokines such as CCL2, CCL19 augment gp120-induced actin dynamics in resting CD4 T cells, which greatly facilitate HIV DNA synthesis and nuclear migration in resting T cells [17,19]. In addition, our results are aligned with a recent study demonstrating that the N-terminal fragment of the Slit2 protein inhibits X4 and R5 viral infection by binding to the Robo1 receptor and antagonizing the HIV gp120-mediated Rac1-LIMK-cofilin pathway for actin rearrangement [78]. Similar interference of the HIV-mediated actin pathway has also been reported in cannabinoid receptor 2-mediated inhibition of X4 viral infection of primary blood CD4 T cells [79]. Nevertheless, genistein can inhibit multiple cellular kinases, and we did not exclude that the inhibition of HIV replication by genistein is a combined effect with the inhibition of actin signaling.

HIV infection is a chronic disease that requires life-long treatment on a daily basis. Thus, persistent and direct inhibition of actin dynamics through actin inhibitors may ultimately cause severe cytotoxicity. However, drug-mediated interference or diversion of HIV-dependent signal transduction to actin activity is achievable. It is our interest to search for inhibitors that are capable of interfering with virus-mediated actin activity while minimally affecting cellular actin dynamics. It is possible that such inhibitors may not drastically diminish HIV replication in a short-term. However, with long-term treatment and possibly lower viral drug-resistance, persistent depression of viral loads could be achievable. Genistein has the potential to be one of these cellular protein-based anti-HIV drugs with a favorably low cytotoxicity.

## Conclusions

In conclusion, in our study, we demonstrated that genistein inhibits HIV infection of CD4 T cells and macrophages. These results may suggest that similar naturally-occurring kinase inhibitors, with little or no-detectable cytotoxicity, may be good candidates for long-term management of HIV infection. Such cellular protein-based therapy may also have additional advantages in combating HIV drug-resistance.

## Methods

### Isolation of resting CD4 T cells and memory CD4 T cells from peripheral blood

Resting CD4 T cells were purified from peripheral blood by two rounds of negative selection as previously described [52]. All protocols involving human subjects were reviewed and approved by the George Mason University IRB. Briefly, for the first round of depletion, we used monoclonal antibodies against human CD14, CD56 and HLA-DR, DP, DQ (BD biosciences). For the second round of depletion, we used monoclonal antibodies against human CD8, CD11b and CD19 (BD biosciences). To purify resting memory CD4 T cells, we used antibodies against human CD8, CD11b, CD19 and CD45RA (BD biosciences) for the second round depletion. Antibody bound cells were depleted by using Dynabeads Pan Mouse IgG (Invitrogen). Purified cells were cultured in RPMI-1640 medium supplemented with 10% heat-inactivated fetal bovine serum (Invitrogen), penicillin (50 U/ml) (Invitrogen), and streptomycin (50 μg/ml) (Invitrogen). Cells were rested overnight before infection or treatment.

### Differentiation of macrophages from peripheral blood monocytes

Macrophages were differentiated from human monocytes from the peripheral blood of HIV-1 negative donors as previously described [80]. Briefly, two million peripheral blood mononuclear cells were plated into each well of six-well plates in serum free RPMI medium for one hour. Adherent cells were cultured in RPMI plus 10% heat inactivated fetal bovine serum (FBS) with 10 ng/ml macrophage colony stimulating factor (M-CSF) (R&D System) for two weeks with medium change for every two days.

### Virus preparation and infection of T cells and macrophages

Virus stocks of HIV-1_NL4-3_[81] and THRO.c/2626 [53] were prepared by transfection of 293T cells with cloned proviral DNA as described [52]. Supernatant was harvested at 48 hours and filtered through a 0.45 μm nitrocellulose membrane. Virus titer (TCID_50_) was measured by infection of a Rev-dependent indicator cell line, Rev-CEM [82,83]. For infection of resting CD4 T cells, 10^3.5^ to 10^4.5^ TCID_50_ units of HIV-1 were used to infect 10^6^ cells. For infection, CD4 T cells were pretreated with genistein, herbimycin, 8-Br-cAMP, or 8-Br-cGMP, incubated with the virus for 2 hours at 37°C, and then washed twice with medium to remove unbound virus and inhibitors. Infected cells were resuspended into fresh RPMI-1640 medium supplemented with 10% heat-inactivated fetal bovine serum, penicillin (50 U/ml), and streptomycin (50 μg/ml) at a density of 10^6^ per ml and incubated for 5 days without stimulation. Cells were activated at day 5 with anti-CD3/CD28 magnetic beads at 2 to 4 beads per cell. For the viral replication assay, 10% of infected cells were taken at days 1, 3, 5, 6, 7, 8, and 9 post infection.

For HIV infection of macrophages, cells were pretreated with genistein (10 μg/ml) or DMSO (1%) for 1 hour, infected at 37°C for 2 hours. Infected cells were washed three times and continuously cultured in RPMI plus 10% FBS without M-CSF. Fresh medium was added every two days. Viral replication was monitored by harvesting supernatant. Levels of p24 in the supernatant were measured using Perkin Elmer Alliance p24 antigen ELISA Kit (Perkin Elmer). Plates were kinetically read using an ELx808 automatic microplate reader (Bio-Tek Instruments) at 630 nm.

### SIV infection and genistein treatment of rhesus macaques

Three rhesus macaques of Chinese-origin (*Macaca mulatta*) were used. All animals were housed at the Tulane National Primate Research Center (TNPRC) and maintained in accordance with the standards of the American Association for Accreditation of Laboratory Animal Care and the “Guide for the Care and Use of Laboratory Animals” prepared by the National Research Council. All studies were reviewed and approved by the Tulane Institutional Animal Care and Use Committee (IACUC). All animals were in the chronic phase of SIVmac251 infection with the plasma viral loads in between 10^2^ to 10^4^ copies/ml. Each animal received 10 mg/kg of genistein daily for 12 weeks by oral administration.

### Quantification of plasma viral RNA in infected rhesus macaques

Real-Time PCR was performed by the Pathogen Detection and Quantitation Core (PDQC) of Tulane National Primate Research Center. Plasma samples were spiked with armored RNA (aRNA; Asurgen, Austin, TX) and centrifuged at 25,000 x g for 1 hour. Viral RNA (vRNA) was extracted from the pellet with Proteinase K (2.5 mg/ml; Life Tech, Carlsbad, CA) and the High Pure Viral RNA kit (Roche Indianapolis, IN). Eluted vRNA (100 μl) was then subjected to the RNA Clean and Concentrator kit (ZYMO Research, Irvine, CA) and eluted in 50 μl from which 15 μl was reverse transcribed using MultiScribe™ Reverse Transcriptase (Life Tech) in a 50-μL gene specific reaction. Fourteen microliters of cDNA was added to TaqMan gene expression master mix (Life Tech) along with primers and probe targeting the *gag* region of SIVmac239 and subjected to 40 cycles of qPCR analyses. Fluorescence signals were detected with an Applied Biosystems 7900HT Sequence Detector. Data were captured and analyzed with Sequence Detector Software (Life Tech). Viral copy numbers were calculated by plotting CT values obtained from samples against a standard curve generated with *in vitro*-transcribed RNA representing known viral copy numbers (and controlled by addition of known copies of aRNA). The limit of detection of the assay is 119 copies per ml of plasma.

### Pre-treatment of cells with signaling pathway inhibitors

Unless specified, cells were pre-treated with pertussis toxin (Sigma) (100 ng/ml), genistein (Calbiochem) (1 μg/ml or 3.7 μM) or herbimycin (Sigma) (1 μM) for 1 hour at 37°C, or with 8-Br-cAMP or 8-Br-cGMP (Sigma) (100 μM) for 15 minutes at room temperature, and then infected with HIV.

### Chemotaxis assay

For chemo-attractant assay, one half million resting CD4 T cells were suspended into 100 μl of RPMI-1640 medium, and then added to the upper chamber of a transwell plate (6.5 mm diameter and 5 μm pore size with a polycarbonate membrane) (Corning). The lower chamber was filled with 600 μl of medium premixed with SDF-1 (R&D systems). The transwell plate was incubated at 37°C for 2 hours, and then the upper chamber was removed and cells in the lower chamber were counted in a Beckman coulter Z2 cell and particle counter.

### FITC-Phalloidin staining of F-actin and flow cytometry

One million cells pretreated with genistein (3.7 μM) for 1 hour at 37°C were stimulated with SDF-1 or HIV-1 for various periods of time. Cells were incubated at 37°C in an Eppendorf Thermomixer with gentle agitation (600 rpm) to prevent cells settling at the bottom. F-actin staining using FITC-labeled phalloidin (Sigma) was carried out according to the manufacturer’s recommendation with minor modifications. Briefly, cells were pelleted, fixed and permeabilized with CytoPerm/Cytofix buffer (BD Biosciences) for 20 minutes at room temperature, washed with cold Perm/Wash buffer (BD Biosciences) twice, followed by staining with 5 μl of 0.3 mM FITC-labeled phalloidin (Sigma) for 30 minutes on ice in the dark. After washing twice with cold Perm/Wash buffer, cells were resuspended in 1% paraformaldehyde and analyzed on a FACSCalibur (BD Biosciences).

### Nuclear DNA fractionation and real time PCR measurement of HIV DNA

Infected cells were directly lysed in DNA extraction lysis buffer (SV Genomic DNA Isolation System, Promega). Total cellular DNA was extracted and viral total DNA was measured by real time PCR as previously described [12]. The fractionation of viral DNA in memory T cells was conducted as previously described [12]. Briefly, cells were pelleted at 270 x *g* for 5 minutes in a microfuge at 4°C, washed once with ice-cold PBS, resuspended into ice-cold cell lysis buffer (10 mM Tris-Cl, pH 7.5, 140 mM NaCl, 5 mM KCl, 1% EDTA, 1% NP-40), incubated on ice for 5 to 10 minutes, and then centrifuged at 270 x *g* for 5 minutes at 4°C to pellet the nuclei. The nuclear pellet was washed once with ice-cold cell lysis buffer and then dissolved in DNA extraction lysis buffer (SV Total RNA Isolation System, Promega). Total cellular DNA was extracted and viral DNA was measured by real time PCR as previously described [12].

### HIV entry assay

The Nef-luciferase-based HIV entry assay was performed as described [56]. Briefly, cells (1 × 10^6^) were infected with 200 ng of Nef-luciferase containing viruses at 37°C for 2 hours, and then washed three times with medium. Cells were resuspended in 0.1 ml of luciferase assay buffer (Promega) and luciferase activity was measured in live cells using a GloMax-Multi Detection System (Promega).

### Western blot to detect LIMK and cofilin activation

One million cells were lysed in NuPAGE LDS Sample Buffer (Invitrogen) and separated by SDS-PAGE, and then transferred onto nitrocellulose membranes (Invitrogen). The blots were washed, blocked with Starting Block blocking buffer (Pierce), and incubated overnight with rabbit polyclonal antibodies specific for phospho-LIMK1/2 (1:1000 dilution) or phospho-cofilin (ser3) (1:500 dilution) (Cell Signaling). The blots were washed and then incubated with goat anti-rabbit 800cw labeled antibodies (Li-cor Biosciences) (1:5000 diluted) for 1h at 4°C. The blots were washed three times and scanned with Odyssey Infrared Imager (Li-cor Biosciences). The same blots were also probed with goat anti-GAPDH antibodies (Abcam, ab9483) (1:1000 dilution). The secondary antibody staining was performed using 1:5000 dilution of Rabbit Anti-Goat IgG DyLight 680 antibodies (KPL). The blots were imaged on an ODYSSEY Infrared imager (Li-Cor Biosciences).

### Conjugation of antibodies to magnetic beads and stimulation of resting CD4 T cells

Monoclonal antibodies against Human CD3 (clone UCHT1), CD28 (clone CD28.2), CD4 (clone PRA-T4) or CXCR4 (clone 12G5) were from BD Biosciences. Antibodies were conjugated to magnetic beads and used to stimulate resting CD4 T cells as previously described [12].

### Confocal Microscopy

Stained cells were imaged using a Zeiss Laser Scanning Microscope, LSM 510 META, with a 40 NA 1.3 or 60 NA 1.4 oil DIC Plan-Neofluar objective. Samples were excited with a laser line, 488 nm for FITC. Images were simultaneously recorded in two channels: channel one, fluorescent emissions from 505 to 530 nm for FITC (green); channel two, DIC. Images were processed and analyzed by the LSM 510 META software.

## Competing interests

The authors declare that they have no competing interests.

## Authors’ contributions

JG and AY carried out T cell chemotaxis, HIV infection, flow cytometry, and inhibitor studies. XX performed confocal microscope imaging and data analysis on chemotactic assays. TR and JG performed experiments on HIV infection of macrophages. AY, DY, HL, FY, and TH performed real time PCR, p24 ELISA, and flow cytometry. YW and BL designed experiments on animal studies, and BL supervised animal studies. YW, JT, JG, and XX designed experiments on chemotaxis assay and performed data analysis. YW conceived and coordinated the study, and drafted the manuscript. All authors read and approved the final manuscript.

## Supplementary Material

Additional file 1: Figure S1 Genistein inhibits HIV infection of resting CD4 T cells. **Figure S2.** Effects of genistein on T cells activation. **Figure S3.** Genistein interferes with SDF-1-mediated actin dynamics in resting memory CD4 T cells. **Figure S4.** Confocal microscopy quantification of the cellular F-actin intensity in resting CD4 T cells. **Figure S5.** Genistein interferes with HIV-mediated actin dynamics in resting memory CD4 T cells. Click here for file
