# Supplementary material for: Genistein interferes with SDF-1- and HIV-mediated actin dynamics and inhibits HIV infection of resting CD4 T cells
Source: Retrovirology. 2013 Jun 19;10:62. doi: 10.1186/1742-4690-10-62 (PMC3693989; doi:10.1186/1742-4690-10-62)
Supplement: Additional file 1: Figure S1 — Genistein inhibits HIV infection of resting CD4 T cells. Figure S2. Effects of genistein on T cells activation. Figure S3. Genistein interferes with SDF-1-mediated actin dynamics in resting memory CD4 T cells. Figure S4. Confocal microscopy quantification of the cellular F-actin intensity in resting CD4 T cells. Figure S5. Genistein interferes with HIV-mediated actin dynamics in resting memory CD4 T cells. [file 1742-4690-10-62-S1.doc]

**Additional file 1:**

**Genistein interferes with SDF-1- and HIV-mediated actin dynamics and inhibits HIV infection of resting CD4 T cells**

Jia Guo^1^, Xuehua Xu^2^, Taban K. Rasheed^1^, Alyson Yoder^1^, Dongyang Yu^1^, Huizhi Liang^1^, Fei Yi^1^, Todd Hawley^1^, Tian Jin^2^, Binhua Ling^3^, and Yuntao Wu^1^ *

^1^National Center for Biodefense and Infectious Diseases, Department of Molecular and Microbiology, George Mason University, Manassas, VA 20110, USA

^2^Chemotaxis Signal Section, Laboratory of Immunogenetics, National Institute of Allergy and Infectious Disease, NIH, Twinbrook Facility, Rockville, MD, 20852, USA

^3^Division of Comparative Pathology, Tulane National Primate Research Center, Tulane University Health Sciences Center, Covington, LA 70433, USA


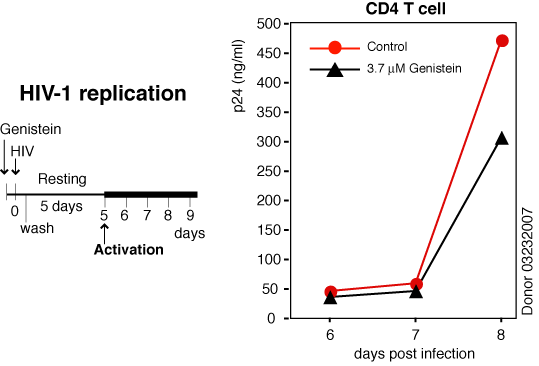


**Figure S1 – Genistein inhibits HIV infection of resting CD4 T cells**

Resting CD4 T cells were pretreated with genistein (3.7 μM) or DMSO (1%, control), and infected with HIV-1_NL4-3_ for 2 hours at 37°C. Following infection, cells were washed twice and then cultured in the absence of the inhibitors for 5 days. Cells were activated at day 5 with anti-CD3/CD28 magnetic beads (4 beads per cell), and viral replication was measured by p24 release. .

**
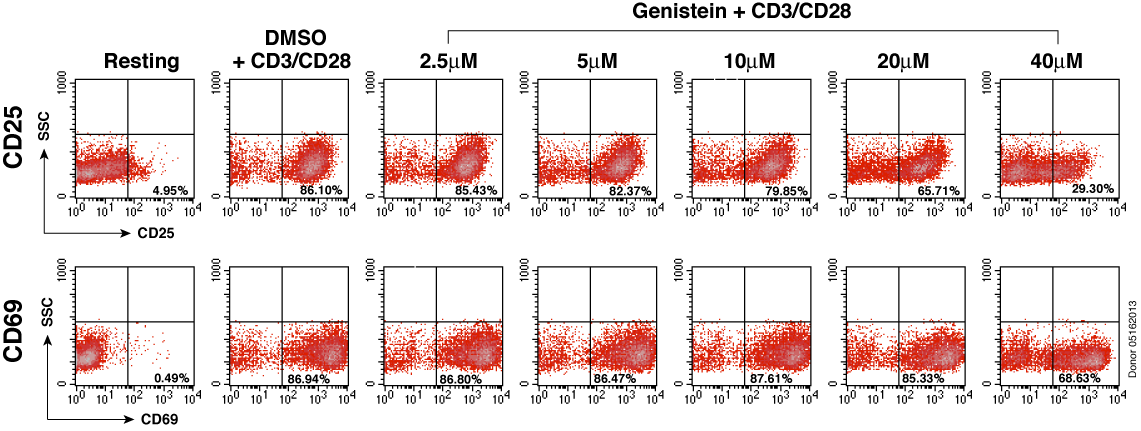
**

**Figure S2 – Effects of genistein on T cells activation.**

A half million resting CD4 T cells were treated with genistein at different dosages, and cultured in the continuous presence of genistein for 2 days, and then activated with anti-CD3/CD28 beads (4 beads per cell). At 24 hours after stimulation, cells were stained with PE-labeled monoclonal antibody against human CD25 (clone RpA-T4) or CD69 (clone 12G5) (BD Biosciences). Cells were stained on ice in PBS + 0.1% BSA for 30 minutes, washed with cold PBS-0.1% BSA, and then analyzed on a FACSCalibur (BD Biosciences). Isotype staining not shown.


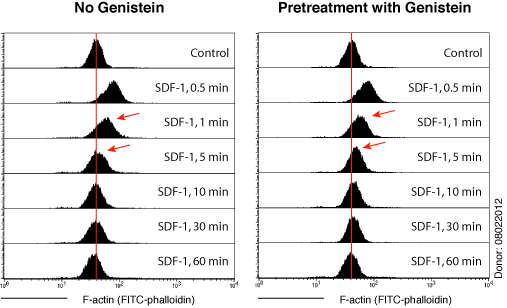


**Figure S3 - Genistein interferes with SDF-1-mediated actin dynamics in resting memory CD4 T cells**

Resting memory CD4 T cells one additional donor were pretreated with genistein (3.7 μM) or mock-treated for 1 hour at 37°C, and then stimulated with SDF-1 (12.5 nM) for various times, from 0.5 minute to 60 minutes. Cells were fixed, stained with FITC-phalloidin for F-actin, and then analyzed with flow cytometer.


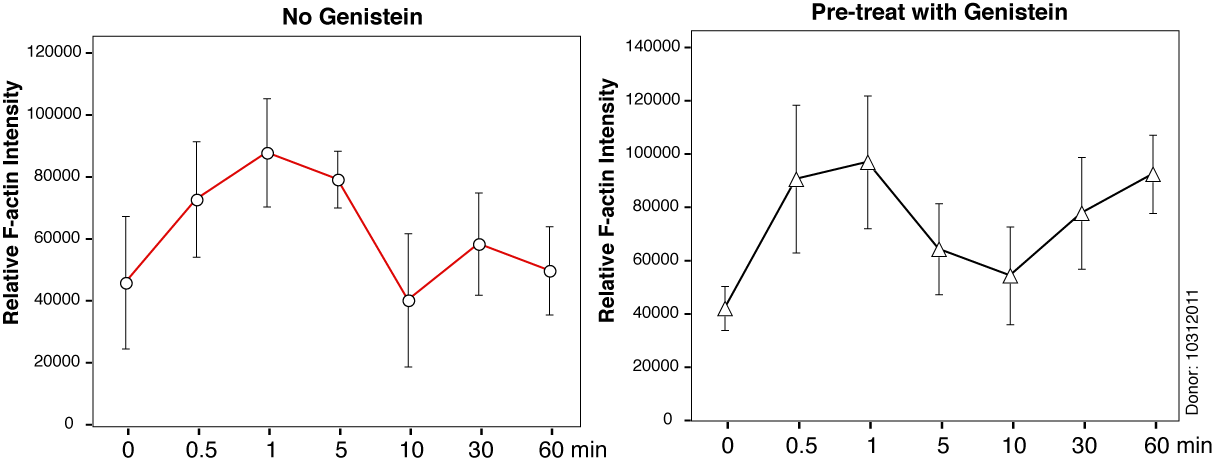


**Figure S4 – Confocal microscopy quantification of the cellular F-actin intensity in resting CD4 T cells.**

Intensity analysis of the confocal images shown in **Figure 4B** was performed using MetaMorph. After converted from original 12 bit to 16 bit, the basal threshold of images was set at 65. The average intensity of cells in each frame were measured and exported to text format, and then plotted.


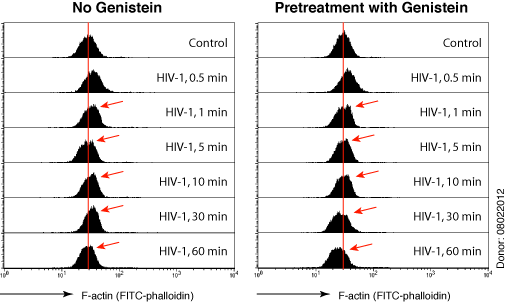


**Figure S5 - Genistein interferes with HIV-mediated actin dynamics in resting memory CD4 T cells**

Resting memory CD4 T cells from two donors were pretreated with genistein (3.7 μM) or mock-treated for 1 hour at 37°C, and then stimulated with HIV-1_NL4-3_ (100 ng) for various times. Cells were fixed, stained with FITC-phalloidin for F-actin, and then analyzed with flow cytometer.
